# Beyond the surface of capacity building: a mixed-methods study of the core functions and forms of dissemination and implementation science consultations

**DOI:** 10.1186/s43058-025-00775-0

**Published:** 2025-08-18

**Authors:** Kera N. Swanson, Nicole A. Stadnick, Gregory A. Aarons, Lauren Brookman-Frazee, Isaac Bouchard, Zeying Du, Anna G. Brubaker, Carrie Geremia, Kelli Cain, Lilliana R. Conradi, Marisa Sklar, Clare Viglione, Borsika A. Rabin

**Affiliations:** 1 Dissemination and Implementation Science Center, UC San Diego Altman Clinical and Translational Research Institute, University of California San Diego, La Jolla, CA USA; 2 San Diego Center for AIDS Research Implementation Science Hub, University of California San Diego, La Jolla, CA USA; 3 UC San Diego ImplementatioN Science and Team Effectiveness in Practice (IN STEP) Children’s Mental Health Research Center, University of California San Diego, La Jolla, CA USA; 4 Department of Psychiatry, University of California San Diego, La Jolla, CA USA; 5 Child and Adolescent Services Research Center, San Diego, CA USA; 6 California State University Northridge, Northridge, CA USA; 7 Herbert Wertheim School of Public Health and Human Longevity Science, University of California San Diego, La Jolla, CA USA; 8 Department of Medicine, University of California San Diego, La Jolla, CA USA

**Keywords:** Dissemination, Implementation, Capacity Building, Consultation

## Abstract

**Background:**

The number of Dissemination and Implementation Science (DIS) capacity building programs is increasing worldwide. These programs aim to enhance diverse DIS skills through a variety of activities. Our team’s systematic review of DIS programs determined that DIS consultations were offered across 67% of programs, yet their specific roles in capacity development were not well defined. This mixed methods study aimed to identify and categorize the functions and forms of consultation activities across three DIS capacity building programs at the University of California San Diego that varied in content focus and infrastructure and then to map findings onto DIS competencies.

**Methods:**

Consultation notes from the three programs were extracted for content analysis to identify discussion topics, DIS guidance provided, and resources shared. Generative artificial intelligence (ChatGPT Plus) facilitated content analyses with multiple rounds of validation from program consultants to refine and finalize themes. Themes were categorized into consultation functions and forms. Forms were mapped onto DIS competencies and a gap analysis was conducted to identify areas for improvement. Program metrics were used to further contextualize findings.

**Results:**

A total of 108 consultation notes were analyzed across the three programs. The most common types of support requested related to grant proposals (54%) and ongoing D&I projects (25%). Consultation discussion topics most frequently involved applying implementation science principles (55%) grant development (31%), guidance centered on DIS methods (47%), and study/project design (37%). Consultation guidance was most often aligned with intermediate-level DIS competencies (58%), primarily in the design and analysis and practice-based considerations domains.

**Conclusions:**

These findings highlight the nature of DIS consultation services, particularly among those seeking support for grant proposals and ongoing implementation projects. Consultations primarily addressed intermediate-level competencies within the domains of design, analysis, and practice-based implementation strategies, indicating a clear opportunity to strengthen support for both foundational and advanced skill development. These results suggest the need for scalable consultation frameworks, improved tracking systems, and tiered training resources to optimize the reach and impact of DIS capacity-building efforts.

**Supplementary Information:**

The online version contains supplementary material available at 10.1186/s43058-025-00775-0.

Contributions to the literature

- Despite the widespread use of consultation in Dissemination and Implementation Science (DIS) capacity-building programs, little is known about what actually happens during these consultations.
- This study identifies the core functions (topics discussed) and forms (guidance provided) of DIS consultations across three DIS capacity-building programs.
- By mapping consultation activities onto established DIS competencies, this work provides a new method for evaluating how consultations support capacity-building efforts.
- Findings highlight opportunities to better align consultation services with foundational DIS competencies, helping to shape future training and consultation models.

## Background

Interest in Dissemination and Implementation Science (DIS) and its applications continues to grow, with a variety of efforts emerging to support its advancement [1, 2]. DIS capacity building programs enhance DIS skills and competencies through education and training, funding opportunities, consultation, technical assistance, and mentorship [3–12].

Notable programs include the Implementation Research Institute (IRI), which provides intensive training in mental health DIS through a two-year program of didactic sessions, mentored research, and concept paper development [13–15]; the Mentored Training for Dissemination and Implementation Research in Cancer (MT-DIRC), targeting postdoctoral researchers with mentorship and project-based learning [16]; the Knowledge Translation Summer Institute (KTSI) in Canada, offering short-term, intensive training on implementation frameworks and stakeholder engagement [17]; and the Training in Dissemination and Implementation Research in Health (TIDIRH), providing foundational training for health researchers in conducting D&I studies [9, 18]. These initiatives reflect a growing ecosystem of DIS training that varies in format (e.g., workshops, fellowships, graduate-level courses etc.), audience, and disciplinary focus [7]. Programs are grounded in DIS competencies, which guide development, assessment, and strengthening of DIS skills [19, 20].

In 2015, Padek and colleagues developed 43-core DIS competencies, organized by level of expertise (i.e., beginner, intermediate, and advanced) and across four domains: (1) Definition, Background, and Rationale; (2) Theory and Approaches; (3) Design and Analysis; and (4) Practice-Based Considerations. Hubeschmann et al. (2022) later expanded these with 10 new competencies aimed at advancing rapid and inclusive DIS research. Grounding capacity-building efforts in these competencies enables rigorous standards and measurable benchmarks for DIS training and workforce development.

Our team conducted a systematic review of DIS capacity building programs, identifying 165 programs domestically and globally, and found that DIS consultations made up 67% of capacity building activities [12]. This highlights the central role of consultation as a strategy for providing tailored guidance, problem-solving, and real-time support to navigate foundational and complex DIS challenges. Despite their prevalence, little is known about how consultation activities contribute to DIS capacity building nor how they align with DIS competencies.

Understanding DIS consultation content and methods is crucial for identifying best practices and developing evidence-based guidelines [21, 22]. Without such knowledge, evaluating, refining, and optimizing consultations remains challenging. Building on prior work [7, 12, 21], this study examines consultation activities at three DIS capacity building programs housed at the University of California, San Diego (UCSD): (1) a DIS program within a clinical and translational science center (NIH UL1TR001442); (2) an HIV implementation science program (NIAID P30-AI036214 CFAR Supplement); and (3) a center focused on team-based implementation strategies in children’s mental health (NIMH P50MH126231). Using consultation notes and program metrics, we aim to identify the core functions (topics) and forms (guidance provided) of DIS consultations, assess their alignment with DIS competencies, and propose best practices to enhance their effectiveness.

## Methods

### Study context: overview of the three DIS capacity building programs

UCSD currently houses three DIS capacity building programs that served as data sources for this study.

1. *The Altman Clinical and Translational Research Institute Dissemination and Implementation Science Center (DISC)* – Established in 2020 through the Clinical and Translational Science Award program (NIH Grant UL1TR001442), offers DIS training, technical assistance, and research support through expert consultations tailored to diverse stakeholders.
2. *San Diego Center for AIDS Research Implementation Science Hub (SD CFAR IS Hub)* – Funded by the National Institute of Allergy and Infectious Diseases (NIAID P30-AI036214 CFAR Supplement) and established in 2020, it is one of 10 regional IS Hubs that supports Ending the HIV Epidemic (EHE) research through DIS training, mentoring, and technical assistance.
3. *Implementation Science and Team Effectiveness in Practice (IN STEP) Children’s Mental Health Research Center* – Established in 2022 through a National Institute of Mental Health (NIMH) ALACRITY P50 award (P50MH126231), IN STEP develops and tests team-based implementation strategies to improve children's mental health services across systems and provides consultation in team effectiveness, implementation science, and children’s mental health services research.

Though operating independently, these programs collaborate by sharing resources and consultants, co-hosting events, and referring consultation requests, offering a unique context for examining DIS consultation activities.

Across programs, 23 consultants (20 PhD/MD-level, three Master’s-level Certified Implementation Support Specialists) offer expertise across DIS, public health, HIV/AIDS, gender and sexual minority health, team-effectiveness research, children’s mental health, leadership, policy, and organizational psychology disciplines (DISC *n* = 11; IS Hub *n* = 8; IN STEP *n* = 11). Seven consultants work across the three programs, fostering cross-program synergy.

Consultation mechanisms were conceptualized using the Core Functions and Forms framework, which distinguishes between an intervention’s essential purposes (core functions) and strategies used to achieve them (forms) [23, 24]. In the context of this study, this framework offers a structured lens through which to examine how various consultation activities contribute to DIS capacity building [25].

### Study design

This study employed a multi-stepped mixed-methods approach:

1. *Data Compilation* – Consultation notes were compiled into a database for qualitative coding and analysis. Program metrics (e.g., consultee role, department, experience level) were integrated to enrich interpretation.
2. *Integration of Program Metrics* –Metrics were synthesized with content analysis findings to contextualize consultation activities.
3. *Content Analysis Using Generative Artificial Intelligence (AI)* – A three-step process combined manual coding with validation using a Large Language Model (LLM) (ChatGPT) [26] to systematically identify and categorize consultation core functions (i.e., topics discussed), forms (i.e., types of guidance provided), and resources shared. Results served as the foundation for competency mapping and gap analysis.
4. *Competency Mapping* – Consultation forms were mapped onto updated DIS competencies [21].
5. *Gap Analysis* – Analysis identified competency areas addressed and gaps in consultation support [27].

### Data compilation

Program coordinators reviewed and added consultation notes to the database, excluding entries lacking sufficient detail. The quality and comprehensiveness of consultation notes varied across the three programs, largely due to note-taking not being standardized across programs or consultants. In some cases, program managers joined consultations and helped document key discussion points, while in others, consultants may have captured notes informally or post hoc. These variations informed our decision to exclude notes lacking substantive content and highlight the need for more standardized documentation approaches in future consultation efforts. Specifically, consultation notes with a single vague sentence, offering no insight into the discussion content, topics covered, or type of guidance provided during the session were excluded. In such cases, these notes did not allow for reliable coding or interpretation and were therefore omitted from the dataset to ensure the integrity and depth of the analysis.

### Integration of program metrics

Program metrics across the three programs (e.g., consulting program, type of consultation, consultee role/title, consultee affiliation, consultation follow up data) were synthesized, providing contextual depth to the content analysis.

Consultee and consultation request data were collected through each program’s consultation request system. DISC and IN STEP offer consultation services to a broader audience and capture a range of requested support types. In contrast, the IS Hub provided consultations exclusively to pre-funded projects under the EHE initiative and did not collect comparable support request data due to its consultation scope.

### Content analysis

The content analysis was completed using three consecutive steps:

Step one – Manual Coding. Two trained coders (KS and IB), both with prior experience conducting qualitative analyses and training in DIS, manually assigned consultation functions, forms, and resource types using an iteratively refined codebook. KS led the development of the initial codebook and trained the second coder (IB) through an onboarding session that included a detailed review of the consultation context, coding structure, and example notes. To ensure consistency and reliability, both coders independently coded a subset of consultation notes and met regularly to review discrepancies, discuss code interpretations, and refine definitions as needed. Any unresolved discrepancies were escalated to the broader study team for consensus. This consensus-based approach promoted analytic rigor and enhanced the trustworthiness of the coding framework. Consultation function codes were initially developed using predefined topic areas listed on each program’s consultation request, where consultees select discussion topics. However, several of these predefined categories were broad and lacked specificity (e.g., DIS methods, designing and evaluating implementation strategies, program design etc.). To enhance analytical clarity, consultation notes were reviewed to identify more granular discussion points and targeted sub-codes under these broader themes and finalized by the larger study team. For example, the broad category of “adaptations” was refined to reflect more specific subtopics such as “adaptation of DIS models,” “intervention tailoring,” and “modification of implementation strategies”. The full list of codes is provided in Additional file 1.

Codes for consultation forms and resources shared were generated based on observed content in the notes rather than a predefined list. As with function codes, these were iteratively reviewed and validated by the larger study team to ensure consistency and accuracy. For example, the coding team discussed with the larger study team how to distinguish between “connect with co-investigator” and “identified mentor” when reviewing consultation notes. It was agreed that “connect with co-investigator” would be reserved for projects not associated with K or other career development awards, whereas “identified mentor” would be used exclusively for consultations involving mentorship support tied to early-career development mechanisms (e.g., K awards). In another instance, the broad code “guidance on sustainability” was reviewed and refined to “guidance on sustainability methods”, better reflecting the type of technical support offered (i.e., applying DIS theories, models, and frameworks to sustainability planning and evaluation, methods to address sustainability barriers, identifying adaptations for sustainability etc.).

Step two—Familiarize ChatGPT to Data, using ChatGPT as a tool to facilitate the content analysis, the coding team familiarized ChatGPT to the database, conducting iterative checks to ensure accurate data. ChatGPT was provided with key background information related to the consultation notes (e.g., “*these notes are from DIS consultations…”*); total number of notes in the database; and a copy of the codebook to use as a reference. It was prompted to summarize the dataset and the coding team used their existing knowledge of the data to question any inconsistencies until an accurate summary was produced. For example, ChatGPT initially miscalculated the number of consultation notes due to formatting errors in the coded entries. Specifically, it misinterpreted commas as delimiters for separate notes rather than as separators between multiple codes within a single entry. To correct this, the team issued a refinement prompt clarifying the structure of the database: *“Note: Each row in the dataset represents one individual consultation note. Within each row, codes are separated by commas but do not indicate separate consultation entries. Do not count comma-separated codes as separate notes. Instead, use the row count to determine the total number of consultation notes in the dataset.”.* This clarification enabled ChatGPT to correctly compute the total number of consultations and to produce accurate frequency counts of individual codes. The refinement prompt strategy was used throughout the analysis to ensure consistency between the model’s interpretation and the database structure. This iterative process helped mitigate the risks of hallucinations and response degradation, as noted in other AI-supported qualitative analysis research [28, 29].

Step three – AI Assisted Thematic Synthesis, the coding team developed a structured prompt framework to guide the model’s thematic synthesis [29], which included prompting ChatGPT to: (1) analyze the database using the codebook to organize content into overarching categories and (2) summarize the frequency of each code within those categories. To begin, ChatGPT was given explicit instructions to analyze the full list of consultation codes (categorized by consultation function, form, and resources shared) and group them into higher-level content buckets. For example, the coding team provided the following instruction: “*Using the codebook, group the consultation forms into broader thematic categories. For each category, include the name, a short label, and a list of the individual codes it contains. Then provide the frequency of each code based on the number of times it appears in the dataset. Ensure the categories are mutually exclusive.”*

Using ChatGPT’s interactive design, the coding team asked follow-up questions to gather additional detail and validate responses. If the coding team was unsure about ChatGPT’s categorization, they would escalate it to the larger study team for review and feedback. For example, the overarching category of “implementation science application” was initially called “implementation strategies & feedback”. However, this overarching theme did not capture all of the other items in the category. The larger study team advised the coding team to have ChatGPT update the name of the overarching category to better capture the application of implementation science principles, not just implementation strategies. The coding team proceeded to instruct ChatGPT to avoid overlapping categories and to suggest revised category names if existing labels were too narrow or broad: *“Review the content within the ‘implementation strategies & feedback’ category. Some codes in this group pertain to broader applications of DIS (e.g., implementation outcomes, team science/team effectiveness, dissemination strategies). Propose a more inclusive label for this category that better reflects these elements.”.* Then, ChatGPT was prompted to quantify the data by summarizing how many times each code appeared in the dataset: “*For each category, list the number of times each individual code appears, and then sum the total number of codes within that category. Output the results in a table format*”.

The iterative approach allowed the team to not only leverage the model’s capacity for thematic synthesis but also maintain oversight and analytic accuracy throughout the content analysis process [29, 30].

### Competency mapping

Consultation forms, representing actionable guidance, were mapped to DIS competencies [21] by linking each form to its most relevant domain (e.g., Theory, Design and Analysis, Practice-Based Considerations). For each form, the study team identified the most closely related competency based on its primary focus. Forms addressing theoretical or conceptual issues were mapped to competencies in Section A (Definition, Background, and Rationale) or Section B (Theory and Approaches). Forms related to methodology and study design were mapped to Section C (Design and Analysis), while those emphasizing community engagement or practical applications were assigned to Section D (Practice-Based Considerations). Forms that did not align with a specific competency were categorized under broader DIS competencies and noted accordingly. Multiple rounds of validation occurred with the study team to ensure the accuracy of the competency mapping.

### Gap analysis

The gap analysis was conducted by comparing the DIS competencies from Hubeschmann et al. (2022) with the DIS competencies mapped to the identified consultation forms. Competencies were categorized across the four competency domains. The number and percentage of competencies covered in each domain were used to assess gaps in coverage across consultation forms.

### Consultation outcomes

The DISC and IN STEP programs collect post-consultation outcome data from the consultees through brief surveys (DISC via JotForm and IN STEP via Qualtrics) sent approximately six months after the initial consultation. Since the IS Hub exclusively supports ongoing funded EHE projects, comparable follow-up mechanisms were not in place and therefore excluded from this analysis.

## Results

At the time of analysis, the three programs conducted a total of 302 consultations: The DISC completed 238 consultations (2019–2024), the IS Hub completed 33 consultations (2021–2024), and IN STEP completed 31 consultations (2022–2024). Average monthly consultation rates were calculated based on annual volumes for each program, not total divided by years in operation: DISC averaged 4.1/month, IS Hub 3.6/month, and IN STEP 2/month. Not all consultations had associated notes. Of the 302 consultation meetings, 121 consultation notes were identified, and 108 included in the analysis after excluding entries with insufficient detail (e.g., one-sentence notes lacking content). These comprised of 46 from DISC, 38 from IS Hub, and 24 from IN STEP.

### Program metrics

#### Consultee demographics

Consultations were requested by a diverse group of consultees with overlapping roles: Assistant Professors (*n* = 86, 26%), Professors (*n* = 62, 18%), Associate Professors (*n* = 55, 16%), Postdoctoral Trainees (*n* = 35, 10%), Graduate Students (*n* = 26, 8%), and Clinicians/Healthcare Providers (*n* = 22, 7%). Disciplines included Medicine (*n* = 137, 43%), Public Health (*n* = 68, 21%), Psychiatry (*n* = 48, 15%), and Psychology (*n* = 27, 9%). DISC and IN STEP also collected data on consultee affiliations: most were from academic institutions (*n* = 261, 87%), followed by healthcare systems (*n* = 18, 6%), and community agencies (*n* = 12, 4%) (See Table 1).

**Table 1:** Program metrics by program

**Roles/Titles**^**a**^	**Overall (*N*** **= 336)**		**DISC (*n*** **= 235)**		**IS Hub (*n*** **= 42)**		**IN STEP (*n*** **= 59)**	
Professor ^D, IS, IN^	62	18%	50	21%	6	14%	6	10%
Associate Professor ^D, IS, IN^	55	16%	31	13%	16	38%	8	14%
Assistant Professor ^D, IS, IN^	86	26%	61	26%	10	24%	15	25%
Adjunct Professor ^IS^	3	1%	-	-	2	5%	-	-
Postdoctoral Trainee ^D, IN^	35	10%	22	9%	-	-	13	22%
Graduate Student ^D, IN^	26	8%	25	11%	-	-	1	2%
Clinician/Healthcare Provider ^D, IN^	22	7%	15	6%	-	-	6	10%
Project/Research Scientist ^D, IS, IN^	15	4%	7	3%	1	2%	6	10%
Non-faculty Researcher ^D^	2	1%	2	1%	N/A	-	-	-
Research Staff ^D, IN^	10	3%	6	3%	N/A	-	4	7%
Director^IS^	3	1%	-	-	3	7%	-	-
Other (Associate Dean etc.)^D, IS^	14	4%	14	6%	-	-	-	-
**Degrees**^**a**^	**Overall (*N* = 117)**		**DISC**		**IS Hub (*n* = 40)**		**IN STEP (*n* = 77)**	
PhD/DrPH^IS, IN^	37	32%	-	-	28	70%	9	12%
ScD ^IS^	1	1%	-	-	1	3%	-	-
DrPH^IS^	2	2%	-	-	2	5%	-	-
MD/DO ^IS, IN^	48	41%	-	-	8	20%	40	52%
MPH/MSPH ^IS, IN^	8	7%	-	-	-	-	8	10%
MA/MS^IN^	11	9%	-	-	1	3%	10	13%
BA/BS/BSN^IN^	10	9%	-	-	-	-	10	13%
**Departments**^**a**^	**Overall (*N* = 317)**		**DISC (*n* = 275)**		**IS Hub (*n* = 42)**		**IN STEP**	
Medicine (Includes all sub-specialties) ^D, IS^	137	43%	124	45%	13	31%	-	-
Public Health ^D, IS^	68	21%	58	21%	10	24%	-	-
Psychiatry ^D, IS^	48	15%	45	16%	3	7%	-	-
Psychology ^D, IS^	27	9%	22	8%	5	12%	-	-
Global Health ^IS^	3	1%	-	-	3	7%	-	-
Nursing ^D^	5	2%	3	1%	2	5%	-	-
Social Work ^D, IS^	8	3%	7	3%	1	2%	-	-
Other (Health Systems, Anthropology, Medical Social Sciences etc.) ^D, IS^	21	7%	16	6%	5	12%	-	-
**Experience Levels**^**a**^	**Overall (*N* = 333)**		**DISC**^**1**^** (*n* = 193)**		**IS Hub**		**IN STEP (*n* = 48)**	
Novice ^D, IN^	147	44%	122	63%	-	-	25	52%
Advanced beginner ^D, IN^	104	31%	91	47%	-	-	13	27%
Intermediate ^D, IN^	54	16%	46	24%	-	-	8	17%
Advanced ^D, IN^	28	8%	26	13%	-	-	2	4%
^1^ *Aggregate of two separate measures of DIS experience*								
**Affiliations**^**a**^	**Overall (*N* = 301)**		**DISC (*n* = 207)**		**IS Hub**		**IN STEP (*n* = 74)**	
Academic institutions ^D, IN^	261	87%	204	90%	-	-	57	77%
Healthcare systems ^D, IN^	18	6%	14	6%	-	-	4	5%
Community agency (e.g., Federally qualified health center) ^D, IN^	12	4%	9	4%	-	-	3	4%
County department (e.g., health, education etc.) ^IN^	2	1%	-	-	-	-	2	3%
Other (Private organization etc.) ^IN^	8	3%	-	-	-	-	8	11%

^a^Available metrics by program: D = DISC, IS = IS Hub, and IN = IN STEP

Additionally, through the DISC and IN STEP consultation requests, consultees are able to self-disclose their experience levels with DIS (DISC-only) and TER (IN STEP-only), respectively. The DISC consultation request includes two separate questions that assess DIS experience: (1) “What is your current level of experience with D&I research?” and (2) “What is your current level of experience with applying D&I models or frameworks to study design?”. The response options (novice, advanced beginner, intermediate, advanced) were mutually exclusive within each question, but respondents could select different levels across the two questions. To provide a comprehensive snapshot of consultees’ DIS experience, we aggregated responses across both questions; therefore, some overlap between categories is expected and total percentages exceed 100%. DISC consultees reported varying DIS experience: 63% (*n* = 122) identified as novices (e.g., no prior engagement with DIS activities or frameworks), 47% (*n* = 91) as advanced beginners (e.g., having participated in some DIS training, contributed to a DIS project, or used DIS frameworks once or twice), 24% (*n* = 46) as intermediate (e.g. having engaged in DIS activities and applied frameworks multiple times, they had not led a DIS-focused project or proposal), and 13% (*n* = 26) as advanced (e.g., having led grants or projects with DIS as a central research focus or incorporated DIS frameworks as a core part of study design).

Similarly, among IN STEP consultees, 52% (*n* = 25) identified as novices (e.g., no prior engagement with TER activities), 27% (*n* = 13) as advanced beginners (e.g., having participated in TER training or contributed to a TER project), 17% (*n* = 8) as intermediate (e.g., having engaged in TER activities, but not led a TER-focused project or proposal), and 4% (*n* = 2) as advanced (e.g., having led grants or projects with TER as a central research focus).

#### Type of support requested

Across 302 consultation requests, 329 distinct support types were reported (requests could include multiple types). Most commonly was support related to grant proposals (*n* = 177, 54%) and ongoing D&I projects (*n* = 82, 25%). Additional requests included D&I mentoring and career development (*n* = 44, 13%), support on publications (*n* = 17, 5%), general program inquires (*n* = 4, 1%), other support (e.g., dissertation support) (*n* = 3, 1%), and Ongoing TER research project (*n* = 2, 1%).

Across the three programs, consultations that focused on grant development supported a wide range of funding mechanisms. The most commonly discussed proposals were R-level grants (35%), with R01s alone accounting for 22.9% of all grant mechanisms reviewed. The remaining R-series consultations included R21 (5.6%), R34 (4.2%), and smaller proportions of R24, R33, R36, and R61 mechanisms. K-level career development awards made up 12.1% of consultations, reflecting the prominence of both mid-career and early-career investigators among consultees. Additional mechanisms included F-level fellowships (2.3%), P-level program/project grants (1.9%), U-level cooperative agreements (0.9%), and a small number of G-level grants (0.5%). Notably, 30.4% of consultations involved grant types categorized as “Other,” which included CFAR administrative supplements, pilot awards, internal merit-based grants, and industry-sponsored proposals.

### Content analysis: consultation functions & forms

#### Consultation functions

A total of 370 topic observations were identified across 108 consultation notes (multiple topics per note). The most common functions included implementation science applications (e.g., implementation strategies; applying DIS theories, models, and frameworks; adaptations etc.) (*n* = 201, 55%) and grant development (e.g., study design; metrics & measures; identifying a co-investigator etc.) (*n* = 113, 31%), while topics on analytical methods and evaluation (e.g., data analysis; qualitative/quantitative methods support etc.) (*n* = 31, 8%) and professional development and training (e.g., publications, discussion of program services, general DIS education etc.) (*n* = 25, 7%) were also present. (See Table 2).

**Table 2:** Content analysis results

Topic Discussed (Consultation Functions)	Frequency (*N* = 370)	Percentage (%)
**Implementation Science Application**	201	54%
Implementation strategies	29	8%
Models & frameworks	28	8%
Community and/or stakeholder engagement	27	7%
Implementation outcomes	23	6%
Implementation determinants	23	6%
Implementation Outcomes Crosswalk	16	4%
Sustainability	12	3%
Adaptations	12	3%
Team science/Team effectiveness	11	3%
Program design	7	2%
Dissemination strategies	5	1%
Team echanisms	5	1%
Intervention Selection	3	1%
**Grant Development**	113	31%
Study design	63	17%
Metrics & measures	23	6%
Seeking co-investigator	12	3%
Funding opportunities	9	2%
Future grant proposal	6	2%
**Analytical Methods and Evaluation Support**	31	8%
Data analysis	13	4%
Qualitative methods support	13	4%
Program evaluation	4	1%
Quantitative methods support	1	0.1%
**Professional Development and Training**	25	7%
Publication/presentation	9	2%
General D&I education	8	2%
Seeking mentor	5	1%
Career development	3	1%
**Types of DIS Guidance Provided (Consultation Forms)**	**Frequency (*N* = 262)**	**Percentage (%)**
**Dissemination & Implementation Concepts and Methods**	124	47%
Guidance on selecting, applying, evaluating, and adapting implementation and/or dissemination strategies	29	11%
Guidance on identifying and addressing implementation determinants	21	8%
Use of DIS tools (e.g., implementation logic model; implementation outcomes crosswalk)	17	7%
Guidance on team science methods	12	5%
Guidance on sustainability methods	9	3%
Provided general D&I education (e.g., discussion of D&I topics and concepts, directing towards educational resources)	9	3%
Identify dissemination avenues (e.g., conference presentations)	1	0.40%
Provide guidance on intervention selection	1	0.40%
Guidance on selecting and applying D&I theories, models, frameworks	24	9%
Adapting D&I theories, models, and frameworks	1	0.40%
**Study & Project Guidance**	96	37%
Guidance on study design	56	21%
Feedback on grant	15	6%
Connect with DIS co-investigator	10	4%
Potential funding opportunities	5	2%
Provide feedback on program	5	2%
Feedback on manuscript	3	1%
Feedback on presentation	2	1%
**Community/Stakeholder Engagement Strategies**	28	11%
Conceptualizing and/or operationalizing stakeholder engagement methods	21	8%
Guidance on community-engagement methods	5	2%
Disseminating findings to community and/or stakeholders	1	0.40%
Evaluating community/stakeholder engagement	1	0.40%
**Mentorship/Career Development**	14	5%
Establishing co-investigator/mentorship	6	2%
General career development (e.g., submitting a K award etc.)	5	2%
Identify DIS mentor	3	1%
**Types of Resources Shared**	**Frequency (*N* = 113)**	**Percentage (%)**
Relevant readings (e.g. journal articles, policy brief, grant RFA etc.)	51	45%
Web resource (e.g. D&I models, webtools)	25	22%
Connect with content expert	19	17%
Education and training opportunities (e.g. workshop information, fellowship opportunities etc.)	14	12%
Offer program support (e.g. Letter of Support, data analysis and interpretation)	2	2%

#### Consultation forms

We documented 262 instances of DIS guidance, categorized as consultation forms (not mutually exclusive). The most frequent consultation form was guidance on DIS concepts and methods (*n* = 124, 47%) (e.g., selecting, applying, evaluating, and adapting implementation strategies, identifying and addressing implementation determinants etc.), followed by study and project guidance (*n* = 96, 37%) (e.g., feedback on study design, grant proposals, and making connections with DIS co-investigators). Additional forms included community/stakeholder engagement strategies (*n* = 28, 11%) (e.g., conceptualizing and operationalizing community/stakeholder methods) and mentorship/career development (*n* = 14, 5%) (e.g., agreeing to be listed as a mentor on career development award). (See Table 2).

#### Types of resources shared

Among 109 documented resources, 45% (*n* = 51) were relevant readings (e.g., journal articles, grant examples), 22% (*n* = 25) were DIS-related webtools (e.g., the D&I Models in Health Webtool [31]), 17% (*n* = 19) were connections to content experts, and 12% (*n* = 14) were educational resources. Letters of support were shared in two consultations (2%). (See Table 2).

### Competency mapping & gap analysis

#### Competency mapping

Of the total 54 DIS competencies [21], 25 were mapped to the consultation forms. Eleven forms aligned with multiple competencies, which were recorded in secondary columns (see Table 3). Sub-forms were also captured (e.g., “Use of DIS tools” was broken down into guidance on logic models and outcomes crosswalks). Notably, of the two uncategorized competencies reported by Huebschmann and colleagues (2022), we were able to map one, “Apply theory and strategies from team science to promote team effectiveness in D&I research”, onto the consultation form “Guidance on team science methods”.

**Table 3:** DIS competencies mapped

Consultation Form	Primary			Secondary		
	**Competency Section**	***Competency***	**Competency Level**	**Competency Section**	**Competency**	**Competency Level**
1. Guidance on selecting, applying, evaluating, and adapting implementation and/or dissemination strategies	Section D: Practice-Based Considerations	Identify and develop sustainable partnerships for D&I research	Intermediate	Section D: Practice-Based Considerations	Use evidence to evaluate and adapt D&I strategies for specific populations, settings, contexts, resources, and/or capacities	Advanced
1a. Guidance on evaluation methods	Section C: Design and Analysis	Identify and measure outcomes that matter to stakeholders, adopters, and implementers	Intermediate	Section C: Design and Analysis	Identify common D&I measures and analytic strategies relevant to your research question(s)	Beginner
1b. Guidance on documenting and evaluating adaptations	Section D: Practice-Based Considerations	Describe how adaptations will be documented throughout the D&I research project	Intermediate	Section D: Practice-Based Considerations	Explain how to maintain fidelity of original interventions during the adaptation process	Intermediate
2. Guidance on identifying and addressing implementation determinants	Section A: Definition, Background, and Rationale	Formulate methods to address barriers and facilitators of D&I research	Intermediate	-	-	-
3. Use of DIS tools (e.g., implementation logic model; implementation outcomes crosswalk)	Not explicitly mapped, but aligns with broader guidance competencies (e.g., Section B: Theory and Approaches; Section C: Design and Analysis). Expanded to include sub-forms that were able to be mapped onto individual competencies					
3a. Guidance on completing/refining implementation outcomes crosswalk	Section B: Theory and Approaches	Characterize process models that support iterative cycles of implementation and adaptation based on learning	-	Section C: Design and Analysis	Develop and assess processes and outcomes that support iterative cycles of implementation and bidirectional flow of information (e.g., learning health systems)	Advanced
3b. Guidance on developing implementation logic model for projects	Section B: Theory and Approaches	Describe a process for designing for dissemination (planning for adoption, implementation, and sustainability during the intervention development stage)	Intermediate	-	-	-
4. Guidance on team science methods	Section B: Theory and Approaches	Apply theory and strategies from team science to promote team effectiveness in D&I research	Beginner	-	-	-
5. Guidance on sustainability methods	Section C: Design and Analysis	Effectively integrate the concepts of sustainability/sustainment and the rationale behind them in D&I study design	Intermediate	Section C: Design and Analysis	Evaluate and refine innovative scale-up and spread methods (e.g., technical assistance, interactive systems, novel incentives, and 'pull' strategies)	Advanced
6. Provided general D&I education (e.g., discussion of D&I topics and concepts, directing towards educational resources)	Section A: Definition, Background, and Rationale	Define and communicate dissemination and implementation (D&I) research terminology	Beginner	-	-	-
7. Identify dissemination avenues (e.g., conference presentations)	Not explicitly mapped to competencies but aligns with broader guidance competencies (e.g., Section D: Practice-Based Considerations)					
8. Provide guidance on intervention selection	Section A: Definition, Background, and Rationale	Determine which evidence-based interventions are worth disseminating and implementing	Intermediate	-	-	-
9. Guidance on selecting and applying D&I theories, models, frameworks	Section B: Theory and Approaches	Identify appropriate conceptual models, frameworks, or program logic for D&I change	Intermediate	Section B: Theory and Approaches	Describe a process for designing for dissemination (planning for adoption, implementation, and sustainability during the intervention development stage)	Intermediate
10. Adapting D&I theories, models, and frameworks	Section D: Practice-Based Considerations	Describe how adaptations will be documented throughout the D&I research project	Intermediate	-	-	-
11. Guidance on study design	Section C: Design and Analysis	Identify and articulate the trade-offs between a variety of different study designs for D&I research	Intermediate	Section C: Design and Analysis	Operationalize hybrid effectiveness-implementation designs when appropriate to accelerate the implementation of evidence-based interventions in real-world settings	Advanced
11a. Guidance on selecting, measuring, and determining implementation outcomes	Section C: Design and Analysis	Identify common D&I measures and analytic strategies relevant to your research question(s)	Beginner	Section C: Design and Analysis	Identify and measure outcomes that matter to stakeholders, adopters, and implementers	Intermediate
11b. Review and provide feedback on measures	Section C: Design and Analysis	Describe gaps in D&I measurement and critically evaluate how to fill them	Advanced	-	-	-
11c. Guidance on data collection, analysis, and interpretation	Section C: Design and Analysis	Describe the application and integration of mixed-method (quantitative and qualitative) approaches in D&I research	Intermediate	Section C: Design and Analysis	Apply common D&I measures and analytic strategies relevant for your research question(s) within your model/framework	Intermediate
12. Feedback on grant	Not explicitly mapped to competencies but aligns with broader guidance competencies (e.g., Section C: Design and Analysis & Section D: Practice-Based Considerations)					
13. Connect with DIS co-investigator	Section D: Practice-Based Considerations	Identify and develop sustainable partnerships for D&I research	Intermediate	-	-	-
14. Potential funding opportunities	Indirectly aligns with sustainability and equity strategies in partnerships (e.g., Section D: Practice-Based Considerations)					
15. Provide feedback on program	Not explicitly mapped to competencies but aligns with broader guidance competencies (e.g., Section C: Design and Analysis; Section D: Practice-Based Considerations)					
16. Feedback on manuscript	Not explicitly mapped to competencies but aligns with broader guidance competencies (e.g., Section C: Design and Analysis; Section D: Practice-Based Considerations)					
17. Feedback on presentation	Not explicitly mapped to competencies but aligns with broader guidance competencies (e.g., Section C: Design and Analysis; Section D: Practice-Based Considerations)					
18. Conceptualizing and/or operationalizing stakeholder engagement methods	Section D: Practice-Based Considerations	Integrate strategies within D&I research to facilitate meaningful stakeholder engagement (e.g., shared power, shared decision-making, co-learning)	Beginner	Section D: Practice-Based Considerations	Describe the appropriate process for eliciting input from community-based practitioners for adapting an intervention	Intermediate
19. Guidance on community-engagement methods	Section D: Practice-Based Considerations	Integrate strategies within D&I research to facilitate meaningful stakeholder engagement (e.g., shared power, shared decision-making, co-learning)	Beginner	Section D: Practice-Based Considerations	Summarize the importance of ethically and culturally competent clinical and community-based research in D&I science	Intermediate
20. Disseminating findings to community and/or stakeholders	Not explicitly mapped to competencies but aligns with broader guidance competencies (e.g., Section C: Design and Analysis; Section D: Practice-Based Considerations)					
21. Evaluating community/stakeholder engagement	Section D: Practice-Based Considerations	Integrate strategies within D&I research to facilitate meaningful stakeholder engagement (e.g., shared power, shared decision-making, co-learning)	Beginner	-	-	-
22. Establishing co-investigator/mentorship	Section A: Definition, Background, and Rationale	Describe the range of expertise needed to conduct D&I research (e.g., mixed-methods experience, economic, organizational, policy, clinical)	Intermediate	-	-	-
23. General career development (e.g., submitting a K award etc.)	Not explicitly mapped to competencies but aligns with broader guidance competencies (e.g., Section C: Design and Analysis; Section D: Practice-Based Considerations)					
24. Identify DIS mentor	Section D: Practice-Based Considerations	Identify and develop sustainable partnerships for D&I research	Intermediate	-	-	-

#### Gap analysis

Among the 24 mapped competencies with defined expertise levels, most were Intermediate (*n* = 14, 58%), followed by Beginner (*n* = 5, 21%) and Advanced (*n* = 5, 21%). Most frequently observed competencies came from Section C: Design and Analysis (*n* = 10, 63%) and Section D: Practice-Based Considerations (*n* = 7, 44%).

For example, “Guidance on study design” mapped to “Identify and articulate the trade-offs between a variety of different study designs for D&I research” (Section C, Intermediate). “Guidance on stakeholder engagement” mapped to “Identify and develop sustainable partnerships for D&I research” (Section D, Intermediate). Fewer competencies were observed in Section A: Definition, Background, and Rationale (*n* = 4, 36%) and Section B: Theory and Approaches (*n* = 3, 33%). (see Table 4).

**Table 4:** DIS competency gap analysis

Competency Sections (*N* = 54)	Competencies Observed (*N* = 25, 46%)		Competencies not observed (*N* = 29, 52%)	
	**Specific Competencies Observed (*n* = 4)**	**Level**	**Specific Competencies Not Observed (*n* = 7)**	**Level**
**Section A: Definition, Background, and Rationale (*N* = 11)**	Formulate methods to address barriers and facilitators of D&I research	Intermediate	Examine the importance of rapid research to advance D&I science concepts and directions	Beginner
	Determine which evidence-based interventions are worth disseminating and implementing	Intermediate	Define what is and what is not D&I research	Beginner
	Define and communicate dissemination and implementation (D&I) research terminology	Beginner	Differentiate between D&I research and other related areas, such as efficacy research and effectiveness research	Beginner
	Describe the range of expertise needed to conduct D&I research (e.g., mixed-methods experience, economic, organizational, policy, clinical)	Beginner	Identify the potential impact of disseminating, implementing, and sustaining effective interventions, including assessments of equity and representativeness	Intermediate
			Assess, describe, and quantify (where possible) the context for effective D&I (setting characteristics, culture, capacity, and readiness)	Intermediate
			Identify existing gaps in D&I research	Intermediate
			Identify the potential impact of scaling down (aka de-implementing) an ineffective but often used intervention	Intermediate
	**Specific Competencies Observed (*n* = 3)**	**Level**	**Specific Competencies Not Observed (*n* = 6)**	**Level**
**Section B: Theory and Approaches (*N* = 9)**	Identify appropriate conceptual models, frameworks, or program logic for D&I change	Intermediate	Describe a range of D&I strategies, models, and frameworks	Beginner
			Design strategies to address the multi-level influences of health inequities as it relates to the implementation of an evidence-based intervention	Intermediate
	Describe a process for designing for dissemination (planning for adoption, implementation, and sustainability during the intervention development stage)	Intermediate	Describe the relationships between various organizational dimensions (e.g., climate, culture) and D&I research	Intermediate
			Explain how knowledge from disciplines outside of health (e.g., business, marketing, and engineering) can help inform further transdisciplinary efforts in D&I research	Intermediate
	Characterize process models that support iterative cycles of implementation and adaptation based on learning	Intermediate	Identify and articulate the interplay between policy and organizational processes in D&I	Intermediate
			Identify core elements (effective ingredients) of effective interventions, and recognize risks of making modifications to these	Intermediate
	**Specific Competencies Observed (*n* = 10)**	**Level**	**Specific Competencies Not Observed (*n* = 6)**	**Level**
**Section C: Design and Analysis (*N* = 16)**	Identify and articulate the trade-offs between a variety of different study designs for D&I research	Intermediate	Describe the core components of external validity and their relevance to D&I research	Beginner
	Describe the application and integration of mixed-method (quantitative and qualitative) approaches in D&I research	Intermediate		
	Identify common D&I measures and analytic strategies relevant to your research question(s)	Beginner	Identify possible methods to address external validity in study design reporting and implementation	Intermediate
	Apply common D&I measures and analytic strategies relevant for your research question(s) within your model/framework	Intermediate	List the potential roles of mediators and moderators in a D&I study	Intermediate
	Identify and measure outcomes that matter to stakeholders, adopters, and implementers	Intermediate		
	Operationalize hybrid effectiveness-implementation designs when appropriate to accelerate the implementation of evidence-based interventions in real-world settings	Advanced	Effectively explain and incorporate concepts of de-adoption and de-implementation into D&I study design	Advanced
	Describe gaps in D&I measurement and critically evaluate how to fill them	Advanced		
	Evaluate and refine innovative scale-up and spread methods (e.g., technical assistance, interactive systems, novel incentives, and 'pull' strategies)	Advanced	Effectively integrate the concepts of sustainability/sustainment and the rationale behind them in D&I study design	Intermediate
	Describe how to frame and analyze the context of D&I as a complex system with interacting parts	Intermediate		
	Develop and assess processes and outcomes that support iterative cycles of implementation and bidirectional flow of information (e.g., learning health systems)	Advanced	Incorporate methods of economic evaluation (e.g., implementation costs, cost-effectiveness) in D&I study design	Advanced
	**Specific Competencies Observed (*n* = 7)**	**Level**	**Specific Competencies Not Observed (*n* = 9)**	**Level**
**Section D: Practice-Based Considerations (*N* = 16)**	Identify and develop sustainable partnerships for D&I research	Intermediate	Describe the importance of incorporating the perspectives of different stakeholder groups (e.g., patient/family, employers, payers, healthcare settings, public organizations, community, and policy makers)	Beginner
	Describe how adaptations will be documented throughout the D&I research project	Intermediate	Describe the concept and measurement of fidelity	Beginner
	Summarize the importance of ethically and culturally competent clinical and community-based research in D&I science	Beginner	Articulate the strengths and weaknesses of participatory research in D&I research	Beginner
	Integrate strategies within D&I research to facilitate meaningful stakeholder engagement (e.g., shared power, shared decision-making, co-learning)	Beginner	Determine when engagement in participatory research is appropriate with D&I research	Intermediate
	Use evidence to evaluate and adapt D&I strategies for specific populations, settings, contexts, resources, and/or capacities	Advanced	Identify and apply techniques for stakeholder analysis and engagement when implementing evidence-based practices	Intermediate
			Identify a process for adapting an intervention and implementation strategy prior to and during implementation	Intermediate
	Explain how to maintain fidelity of original interventions during the adaptation process	Intermediate	Identify sites to participate in D&I studies, and negotiate or provide incentives to secure their involvement	Intermediate
	Describe the appropriate process for eliciting input from community-based practitioners for adapting an intervention	Intermediate	Describe how to measure successful partnerships for D&I research	Intermediate
			Develop strategies to promote equity in resource distribution across all external research partners, including community partners or other external organizations and the researcher’s institution	Intermediate
	**Specific Competencies Observed (*n* = 1)**	**Level**	**Specific Competencies Not Observed (*n* = 1)**	**Level**
**Not Categorized (*N* = 2)**	Apply theory and strategies from team science to promote team effectiveness in D&I research	N/A	Apply systems science and systems modeling approaches in D&I research	N/A

### Consultation follow up data

#### Consultation satisfaction

Of the 223 DISC consultation follow up surveys collected, 77 had completed satisfaction questions. Consultees rated their consultation experience using a 5-point Likert scale (1 = Strongly Disagree to 5 = Strongly Agree). Responses were highly positive: 100% agree consultants actively listened (*n* = 77), 93.4% (*n* = 71) strongly agreed the consultation was useful, and 90.9% (*n* = 70) strongly agreed the meeting was timely and that they would recommend the service. Somewhat lower agreement (84.4%, *n* = 65) was seen for post-meeting collaboration follow-through (See Table 5). One consultee shared: *“DISC consultation helped me establish my first connection with a DISC expert, from which a mentor–mentee relationship was developed (and still is). Through this relationship I was able to tailor my K01 proposal with strong Implementation Science focus. The proposal was completed and submitted”.*

**Table 5:** DISC consultation satisfaction ratings

	Strongly Disagree	Somewhat Disagree	Neither Agree/Disagree	Somewhat Agree	Strongly Agree
The consultation meeting was scheduled in a timely manner	-	1 (1.3%)	1 (1.3%)	5 (6.5%)	70 (90.9%)
My immediate needs were addressed during this first consultation	-	1 (1.3%)	-	9 (11.8%)	66 (86.8%)
The consultation meeting was useful	-	-	-	5 (6.6%)	71 (93.4%)
The consultant addressed my questions clearly and completely	-	-	1 (1.3%)	5 (6.6%)	70 (92.1%)
I understand my steps or action items	-	-	1 (1.7%)	7 (11.5%)	53 (86.9%)
There is a clear continuation plan to keep my work moving forward	-	-	3 (3.9%)	12 (15.6%)	62 (80.5%)
Resources or tools mentioned during the consultation were shared afterwards	-	-	1 (1.3%)	7 (9.2%)	68 (89.5%)
The consultation improved my understanding of D&I frameworks and models	-	-	-	6 (12.0%)	44 (88.0%)
The consultant actively listened to my questions and description	-	-	-	-	75 (100.0%)
Connections and/or collaborations offered during the meeting were shared afterwards	-	-	6 (7.8%)	6 (7.8%)	65 (84.4%)
I would recommend the DISC consultation service to my colleagues	-	-	-	7 (9.1%)	70 (90.9%)
The consultation improved my ability to apply D&I frameworks and models	-	-	-	8 (16.7%)	40 (83.3%)

Participants rated each item on a 5-point Likert scale ranging from 1 (Strongly Disagree) to 5 (Strongly Agree). *D&I* Dissemination and Implementation. Data presented as a frequency

For IN STEP, there were a total of 13 follow up surveys collected. All respondents selected the highest possible score across satisfaction with the consultation service (“Extremely satisfied”), perceived value of the consultation (“Very valuable”), and likelihood to recommend the service (“Extremely likely”). Due to uniformity in responses, these data are summarized narratively.

#### Project outcomes

**Fig. 1:**
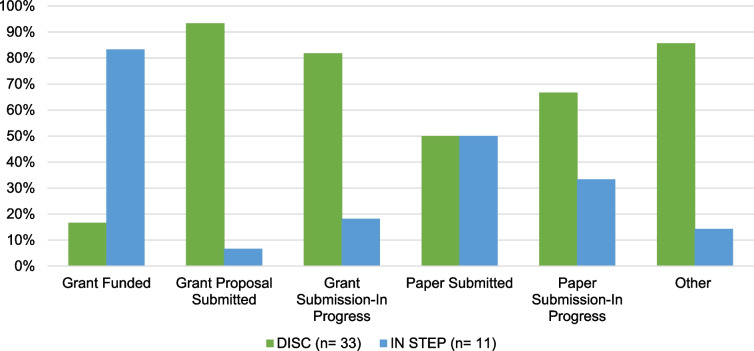
DISC and IN STEP Consultation Outcomes – Project Status. *Note:* Percentages represent the proportion of respondents within each program (DISC: *n* = 33; IN STEP: *n* = 11) who reported each outcome. Raw values are not shown due to different sample sizes across programs

DISC and IN STEP also collected data on project status. Six non-mutually exclusive categories were shared across the two programs: Grant Proposal Submitted (34.1%, *n* = 15), Grant Submission-In Progress (25.0%, *n* = 11), Other (15.9%, *n* = 7), Grant Funded (13.6%, *n* = 6), Paper Submission-In Progress (6.8%, *n* = 3), and Paper Submitted (4.5%, *n* = 2) (See Fig. 1.)

## Discussion

This study expands our understanding of DIS consultations by examining core functions and forms of consultations across three DIS capacity-building programs. While several formal DIS training programs have evaluated educational activities (e.g., coursework, seminars, mentoring), less is known about the consultation services embedded within these programs [3–5, 9, 13–15, 15, 20, 32]. Given that consultations represent approximately 67% of DIS capacity-building activities [12], this study fills a critical gap by detailing consultation content, guidance forms, and alignment with DIS competencies [20]. These findings contribute to a more nuanced understanding of consultations as a central DIS capacity-building mechanism.

Program metrics showed that most consultees were faculty at academic institutions (ranging from early-career to senior investigators) or clinicians/healthcare professionals working in healthcare systems, often identifying as novices. Consultation largely supported ongoing DIS projects and grant proposals. Notably, the IS Hub’s focus on Ending the HIV Epidemic projects likely contributed to its higher proportion of project-based consultations. The range of grant mechanisms addressed through DIS consultations reflects the broad scope of capacity-building needs across career stages and funding pathways. Majority of the grant-related consultations were focused on R01 proposals, indicating strong demand for advanced DIS guidance on large-scale research projects. K-series career development awards and F31 fellowships made up the second most common type of grant mechanism discussed, emphasizing the important role of DIS consultations in supporting early-career investigators integrate DIS principles into training-focused proposals. These findings highlight the dual role of consultations as a both technical and developmental capacity-building strategy.

Follow up data from DISC and IN STEP consultations indicated high levels of satisfaction, with strong ratings across satisfaction dimensions. Project outcomes suggested early signs of consultation impact: 34.1% of DISC and IN STEP consultees reported grant proposal submissions, and 13.6% reported funded grants following consultation. These findings reinforce consultations’ potential value not only for education but also for advancing tangible research outputs [33].

Content analysis systematically identified consultation functions (topics discussed), forms (guidance provided), and resources shared [23–25]. Implementation science applications and grant development emerged as the most common functions; DIS methods guidance and study/project support were the most frequent forms. Shared resources included relevant readings, DIS web tools, expert connections, and educational opportunities. Although letters of support were infrequently shared during sessions, they remain a resource offered by these programs outside of the consultation service and not explicitly captured through consultation notes.

Mapping consultation forms to DIS competencies allowed objective evaluation of how these consultations align with DIS training standards [7, 19–21, 34]. The gap analysis showed concentration in competencies related to Design and Analysis and Practice-Based Considerations, with fewer consultations addressing foundational theory and background. Additionally, consultations were predominantly aligned with intermediate-level competencies, with fewer consultations mapping onto beginner- and advanced-level competencies.

Applying the Core Functions and Forms framework to consultations helped to clarify their role among DIS capacity building efforts [23–25] and offers a framework for evaluation [2, 34]. Identifying gaps in consultation coverage presents an opportunity to enhance training and foundational DIS skill development.

### Limitations

Despite generating valuable insights, this study has limitations. First, while the three programs share administrative resources and commonalities, their distinct structures and processes complicated data synthesis. For example, consultation documentation varied in format and quality across programs. While some consultations were supported by program coordinators who helped document detailed notes, in other cases, notes were entered post hoc by consultants with differing levels of detail. Note-taking responsibilities were not standardized, and the individual responsible for documentation was not always clearly identifiable. This resulted in only 108 of the 302 total consultation meetings (36%) being included in the analysis. While this sample provided rich and diverse data across all three programs, the limited availability of analyzable notes may have constrained the generalizability and comprehensiveness of the findings. Developing systematic methods for note-taking and outcome tracking across programs could improve data comparability, enhance analytical rigor, and capture long-term impacts more effectively. One example of this could involve recording consultations and using AI tools to generate transcripts and meeting summaries to enhance the quality and comprehensiveness of consultation records. This approach could reduce the burden of manual note-taking, ensure consistency, and provide valuable resources for training future consultants. However, AI-generated transcripts and notes must be validated by program coordinators to mitigate inaccuracies. This is because AI transcription tools may struggle with specialized terminology, multiple speakers, or poor audio quality, potentially resulting in incomplete or misleading data [29, 35, 36].

Second, because this study relied on written consultation notes and program tracking metrics, our ability to capture the full nuance of consultant-consultee interactions was also limited. Future studies should consider integrating qualitative interviews, direct observation, and case study approaches to triangulate data sources and generate a more comprehensive understanding of how consultations evolve and influence capacity-building outcomes over time.

Third, although ChatGPT supported the content analysis (organizing codes, summarizing frequencies, proposing categories), all outputs were reviewed and refined by the study team. Clarification was often needed to avoid misclassification due to database formatting or code ambiguities. While this validation minimized AI biases, reliance on AI tools still poses risks of oversimplification or contextual misinterpretation [29, 30]. Future research should continue to explore best practices for integrating generative AI tools into qualitative and mixed methods research, including structured prompts, validation strategies, and mitigation of AI limitations.

Fourth, while this study focused on individual consultation sessions, it is important to note that consultations can be requested multiple times across different projects or phases of work. In this initial iteration of this study, we did not examine repeat consultations or long-term consultant-consultee relationships, but future analyses will explore these patterns and their impact on capacity building.

### Implications for future research and practice

Findings suggest several practical strategies for optimizing DIS consultation services. First, improving consultation tracking and standardization could enhance program evaluation and demonstrate impact. Pre/post surveys, qualitative interviews, and periodic follow ups would provide richer insights into consultation contributions to skill-building and project outcomes. Second, the gap analysis identified lower coverage of competencies related to Definition, Background, and Rationale and Theory and Approaches. Since these domains serve as the foundation for DIS, consultation services could intentionally integrate theoretical and background content into sessions (e.g., onboarding modules for newcomers) or complementary training activities appropriate across career stages and experience levels [2, 3]. All three programs offer ongoing training and learning opportunities beyond consultation services, including educational webinars, mock grant reviews, and other targeted educational supports [37]. DIS Consultations often serve as an entry point to these extended supports. Future work should examine the relationship between one-time and reoccurring consultations, along with subsequent engagement in broader program offerings, contribute to overall DIS capacity-building.

Third, formalizing resource-sharing practices, such as curated repositories of recommended readings and webtools, could streamline consultations [38]. Expanding mentorship networks and expert connections would also strengthen capacity-building efforts [39]. Fourth, these results can inform the development of standardized DIS consultation frameworks that can be adapted and scaled across different institutions [2]. Shared consultation protocols, structured resource sharing, and standardized evaluation indicators would strengthen individual programs and foster a broader DIS infrastructure. Additionally, co-developing these frameworks and shared protocols with both consultants and consultees would provide essential user-centered perspectives, ensuring that resulting models are contextually relevant, feasible to implement, and responsive to the diverse needs of those delivering and receiving DIS support.

Finally, while this study primarily focused on describing the core functions and forms of DIS consultations and mapping them to DIS competencies, future analyses should explore how specific forms of DIS guidance contribute to improving the translation of evidence into practice or promoting health equity. For example, consultation forms emphasizing stakeholder engagement, adaptation of interventions, or team science could be examined for their alignment with theories of equitable implementation. Mapping consultation activities to these emerging theoretical priorities may enhance the strategic orientation of DIS capacity-building programs toward broader system change.

## Conclusion

This study provides foundational insights into the functions and forms of DIS consultations within capacity-building programs. By mapping consultation guidance onto established DIS competencies, we offer a structured approach for evaluating how consultations support skill development and project advancement. While findings suggest several potential strategies, such as standardized protocols, structured resource sharing, and shared evaluation practices, these should be considered exploratory. Future research is needed to test, refine, and co-develop these approaches with stakeholders to ensure their relevance, feasibility, and impact. As DIS consultation services continue to evolve, intentional efforts to evaluate and improve these practices will be critical for building a responsive, scalable, and equitable DIS infrastructure.

## Supplementary Information

Additional file 1.

## Data Availability

The datasets used and/or analyzed during the current study are available from the corresponding author on reasonable request [KS]. The datasets presented in this article are not readily available because the project is still ongoing.

## Abbreviations

DIS: Dissemination and implementation science
IRI: Implementation Research Institute
MT-DIRC: Mentored Training for Dissemination and Implementation Research in Cancer
KTSI: Knowledge Translation Summer Institute
TIDIRH: Training in Dissemination and Implementation Research in Health
DISC: Dissemination and Implementation Science Center
SD CFAR IS Hub: San Diego Center for AIDS Research Implementation Science Hub
IN STEP: Implementation Science and Team Effectiveness in Practice
NIH: National Institutes of Health
NIMH: National Institute of Mental Health
NIAID: National Institute of Allergy and Infectious Diseases
AI: Artificial Intelligence
LLMs: Large Language Models
EHE: Ending HIV Epidemic
TER: Team effectiveness research
D&I: Dissemination and implementation
