# Supplementary material for: Beyond the surface of capacity building: a mixed-methods study of the core functions and forms of dissemination and implementation science consultations
Source: Implement Sci Commun. 2025 Aug 18;6:87. doi: 10.1186/s43058-025-00775-0 (PMC12359906; doi:10.1186/s43058-025-00775-0)
Supplement: Supplementary file 1 — Additional file 1. [file 43058_2025_775_MOESM1_ESM.docx]

**Additional file 1.**

*Codebook* ***-*** *Consultation Functions, Forms, and Types of Resources Shared*

| **Consultation Functions (Topics Discussed)** | |
| --- | --- |
| **Code** | **Description** |
| **Adaptations** | Modifying existing interventions, programs, strategies, or study components to fit a new context, population, or setting, while maintaining core functions. |
| **Models & Frameworks** | Selecting, adapting, or applying dissemination and implementation science models, theories, or frameworks to guide project design or evaluation. |
| **Data Analysis** | Analyzing qualitative or quantitative data, interpreting findings, or selecting appropriate analytic approaches in a DIS context. |
| **Implementation Determinants** | Identification and assessment of contextual factors (e.g., barriers, facilitators) that influence implementation success, often using determinant frameworks (e.g., EPIS and CFIR). |
| **Publication** | Manuscript preparation, selecting target journals, responding to reviewer feedback, or framing DIS elements for publication. |
| **Presentation** | Preparing or structuring presentations for scientific conferences, stakeholder meetings, or grant review panels. |
| **Implementation Outcomes** | Selecting, measuring, or interpreting implementation-specific outcomes (e.g., fidelity, adoption, acceptability, sustainability). |
| **Community and/or Stakeholder Engagement** | Strategies for engaging patients, providers, community partners, or other stakeholders in the design, implementation, or dissemination of a project. |
| **Implementation Strategies** | Selecting, adapting, or operationalizing implementation strategies to improve the uptake, delivery, or sustainability of interventions. |
| **Implementation Outcomes Crosswalk** | Use or adaptation of the Implementation Outcomes Crosswalk tool to align strategies, outcomes, and measurement approaches across project components. |
| **Program Design** | Consultations addressing the structural design of a new program, service, or initiative, including workflows, staffing models, or service delivery processes, relating them to implementation outcomes and strategies. |
| **Study Design** | Formulating study aims, research questions, or methodological approaches within a DIS research context. |
| **Dissemination Strategies** | Identifying, developing, and tailoring strategies to effectively share findings with scientific, clinical, policy, or community audiences. |
| **Seeking Co-Investigator** | Requests to be connected with collaborators or co-investigators with specific DIS or content expertise relevant to a planned grant proposal. |
| **Future Grant Proposal** | Planning for future grant submissions, including developing a DIS-aligned proposal concepts. |
| **Funding Opportunity** | Identification or discussion of specific grant mechanisms or funding announcements aligned with DIS project goals. |
| **Sustainability** | Designing for or assessing sustainability of interventions or implementation strategies over time and across settings. |
| **Team Effectiveness** | Discussions related to team-based implementation processes, including team composition, leadership, collaboration, and functioning. |
| **Qualitative Methods Support** | Qualitative research methods, such as interview/focus group design, coding, or thematic analysis. |
| **Quantitative Methods Support** | Quantitative methods, including survey design, statistical modeling, or power analysis in DIS contexts. |
| **Program Evaluation** | Planning or conducting evaluations of programs or interventions, including formative, process, or outcome evaluation. |
| **General DIS Education** | Introductory-level discussions providing a general overview of DIS concepts, terminology, and resources for learning. |
| **Seeking Mentor** | Requests for mentorship or long-term guidance from DIS experts, particularly for career development or early-stage investigators. |
| **Career Advice** | Broader professional development advice related to building a DIS research trajectory, including networking and job market strategies. |
| **Intervention Selection or Design** | Choosing or developing interventions to be implemented or tested in a DIS research or practice context. |
| **De-Implementation** | Reducing, replacing, or eliminating low-value or ineffective clinical or organizational practices. |
| **Team Mechanisms** | Exploration of mechanisms through which teams operate and influence implementation outcomes, such as psychological safety, communication, or coordination. |

| **Consultation Forms (DIS Guidance Provided)** | |
| --- | --- |
| **Code** | **Description** |
| **Guidance on study design** | Support for structuring the overall design of a DIS study, including selecting study types, formulating research questions, and aligning methods with project aims. |
| **Guidance on data collection, analysis, and interpretation** | Guidance on designing data collection tools, selecting analytical methods (qualitative, quantitative, mixed methods), and interpreting findings within a DIS framework. |
| **Guidance on selecting, applying, evaluating, and adapting implementation and/or dissemination strategies** | Support for choosing, tailoring, and assessing strategies that enhance adoption, implementation, or spread of interventions. |
| **Guidance on selecting and applying DIS theories, models, frameworks** | Assistance in identifying and operationalizing theoretical frameworks to guide implementation planning, evaluation, and interpretation. |
| **Guidance on selecting, measuring, and determining implementation outcomes** | Advice on identifying relevant implementation outcomes (e.g., fidelity, acceptability), selecting validated measures, and integrating them into project design. |
| **Guidance on identifying and addressing implementation determinants** | Support in identifying contextual factors (barriers/facilitators) that affect implementation success, often using determinant frameworks (e.g., EPIS, CFIR). |
| **Conceptualizing and/or operationalizing stakeholder engagement methods** | Help developing systematic approaches for engaging stakeholders across planning, implementation, and dissemination phases. |
| **Feedback on grant** | Review and feedback on draft grant proposals, including DIS sections, conceptual clarity, and alignment with funding mechanisms. |
| **Guidance on completing and/or refining implementation outcomes crosswalk** | Support in applying the Outcomes Crosswalk tool to align strategies with outcomes and determine appropriate measures. |
| **Guidance on team science methods** | Advice on enhancing team functioning, including communication, coordination, and applying team science frameworks in implementation research. |
| **Review and provide feedback on measures** | Evaluation of survey or assessment tools for construct alignment, psychometric properties, and suitability for DIS studies. |
| **Connect with DIS co-investigator** | Assistance in identifying and linking with DIS experts for collaboration on research projects or proposals. |
| **Suggestion for implementation measures** | Recommendations for selecting validated implementation measures aligned with specific outcomes or settings. |
| **Guidance on sustainability methods** | Consultation on frameworks and strategies for designing or evaluating the long-term sustainability of implemented interventions. |
| **Provided general DIS education** | Introductory-level explanation of DIS science concepts and terminology; may include directing consultees to relevant learning resources. |
| **Establishing co-investigator/mentorship** | Support in identifying mentors or potential co-investigators with relevant DIS expertise for research development or career support. |
| **Potential funding opportunities** | Identification or discussion of appropriate funding announcements, mechanisms, or agencies for DIS-related work. |
| **General Career Development** | Guidance on academic and professional development (e.g., applying for K or R-series awards, navigating career pathways in DIS). |
| **Guidance on community-engagement methods** | Recommendations for engaging community partners through culturally responsive and equity-informed approaches. |
| **Provide feedback on program** | Consultant reviews and provides feedback on proposed or existing programs with attention to structure, implementation approach, and DIS alignment. |
| **Identify DIS mentor** | Assistance in identifying a mentor with DIS expertise, often for early-stage investigators or trainees applying for career development awards. |
| **Guidance on evaluation methods** | Support in designing program or implementation evaluations (formative, process, outcome), including logic models and evaluation questions. |
| **Feedback on manuscript** | Review and critique of draft manuscripts with emphasis on structure, clarity, DIS integration, and fit with target journals. |
| **Guidance on documenting and evaluating adaptations** | Advice on tracking and assessing modifications to interventions or strategies while maintaining fidelity to core components. |
| **Guidance on developing implementation logic model for projects** | Support in designing a logic model to visually represent relationships among resources, activities, outputs, outcomes, and strategies. |
| **Feedback on presentation** | Review of slides, messaging, and framing for conference presentations, stakeholder briefings, or other dissemination events. |
| **Disseminating findings to community and/or stakeholders** | Strategies for translating and tailoring research findings for non-academic audiences and community partners. |
| **Adapting DIS theories, models, and frameworks** | Guidance on tailoring existing DIS frameworks to suit a specific context, population, or research stage. |
| **Identify dissemination avenues** | Help identifying appropriate venues (e.g., conferences, community forums) and formats (e.g., policy briefs, infographics) for research dissemination. |
| **Evaluating community/stakeholder engagement** | Support in measuring the quality, process, or outcomes of stakeholder involvement using engagement evaluation tools. |
| **Provide guidance on intervention selection** | Assistance in choosing or refining evidence-based interventions for implementation, aligned with contextual needs and project goals. |

| **Types of Resources Shared** | |
| --- | --- |
| **Code** | **Description** |
| **Relevant readings** | Scholarly articles, grant solicitations (e.g., Request for Applications), policy briefs, or other written materials provided to enhance understanding of specific DIS concepts, methods, or funding opportunities. |
| **Web resources** | Online tools or platforms shared to support DIS practice, such as the D&I Models in Health webtool, strategy repositories, or DIS framework databases. |
| **Connect with content expert** | Referrals or introductions to subject matter experts in areas relevant to the consultee’s project needs (e.g., frameworks, clinical content, community engagement). |
| **Education and training** | Information on formal learning opportunities, including workshops, short courses, webinars, fellowships, and training programs in DIS science. |
| **Dissemination opportunities** | Recommendations for venues to disseminate work, including relevant conferences, calls for papers, or journal outlets aligned with DIS content |
| **Offer program support** | Programmatic services provided beyond consultation, such as drafting letters of support, assisting with data analysis, or contributing to project deliverables. |
